# Closed-loop bowel obstruction caused by a right inguinal hernia containing the transverse colon: an unusual case report

**DOI:** 10.1093/jscr/rjaf081

**Published:** 2025-02-25

**Authors:** Abdulaziz Alnumay, Salman Batais, Alwateen Albalawi, Najd Alzaharani, Saleh Husam Aldeligan

**Affiliations:** Department of Surgery, Division of General Surgery, King Saud University, 12372 Riyadh, Saudi Arabia; Department of Surgery, Division of General Surgery, King Saud University, 12372 Riyadh, Saudi Arabia; Collage of Medicine, King Saud University, 12372 Riyadh, Saudi Arabia; Collage of Medicine, King Saud University, 12372 Riyadh, Saudi Arabia; Collage of Medicine, King Saud University, 12372 Riyadh, Saudi Arabia

**Keywords:** case report, closed-loop bowel obstruction, inguinal hernia, Shouldice repair, transverse colon herniation

## Abstract

Inguinal hernias are common, with a lifetime risk of 25% in men. Approximately 10% become incarcerated, risking complications like strangulation and bowel obstruction. Herniation of the transverse colon (TC) is rare, especially when causing a closed-loop obstruction. A 67-year-old male with poorly controlled type II diabetes and hypertension presented with a symptomatic right inguinoscrotal hernia, showing nausea, vomiting, and partial bowel obstruction. A computed tomography (CT) scan revealed a herniated TC causing a closed-loop obstruction. Emergency open hernia repair with a modified Shouldice technique and mesh was performed. Viable TC and omentum were reduced. The patient recovered without complications and was discharged on the fourth postoperative day. This case involved a rare TC herniation causing a closed-loop obstruction, confirmed by CT imaging. The open approach was selected due to the risk of ischemia and anticipated adhesions. A modified Shouldice repair with mesh reinforced the inguinal canal, reducing recurrence risk. The patient’s postoperative course was uneventful. This case highlights the rarity of TC herniation causing closed-loop obstruction. CT scans were crucial for diagnosis, enabling prompt surgical intervention, which is key to preventing serious complications.

## Introduction

Inguinal hernias are the most common type of hernia in adults, where abdominal contents protrude through the inguinal canal [1]. They are more common in men, with about a 25% lifetime risk of developing an inguinal hernia [2]. Approximately 10% of inguinal hernias become incarcerated rendering them irreducible. If the blood supply to the incarcerated tissue is compromised, the hernia becomes strangulated [3, 4].

Bowel obstruction, which can involve the small or large intestine, accounts for ~15% of hospital admissions for acute abdominal pain. Inguinal hernias contribute significantly to these cases, with ~19.6% presenting with obstruction [5]. Intestinal obstruction can be partial, complete, or closed-loop obstruction which is a particularly dangerous form that occurs when a segment of bowel is blocked at two separate points, leading to a high risk of ischemia and perforation [6]. Causes of obstruction range from adhesions and neoplasms to hernias and inflammatory bowel disease. Small bowel obstructions are more frequent than large bowel obstructions, with large bowel obstruction constituting 10–15% of all bowel obstructions [6, 7].

Past studies have documented the herniation of various organs through the inguinal canal, with the small intestine and omentum being the most common [1]. However, less frequently herniated structures, such as the appendix, urinary bladder, Meckel’s diverticulum, ovary, or the ureter have been observed [1, 8]. Transverse colon (TC) herniation through the inguinal canal is exceedingly rare, with only a few reported cases, none involving a closed-loop obstruction [1].

This report presents a case of closed-loop bowel obstruction as the first presentation of an incarcerated right inguinal hernia containing the TC. This case highlights the importance of considering rare presentations in patients with inguinal hernias and underscores the need for prompt surgical intervention to prevent serious complications. This work has been reported in line with the SCARE criteria [9].

## Case presentation

A 67-year-old male presented to our clinic with a progressively enlarging right inguinal swelling. The patient reported a long-standing hernia for the past 20 years that remained asymptomatic until the last 3 months where he noticed an increase in the swelling’s size and difficulty ambulating. The swelling occasionally becomes irreducible and gradually descended into the right scrotum. During the week prior to his clinic visit, the patient experienced symptoms suggestive of partial bowel obstruction, including nausea, vomiting, and diarrhea.

The patient has a history of type II diabetes mellitus managed with insulin, which was poorly controlled with the last HbA1C recorded at 9.6 and also suffers from hypertension. He had no other comorbidities and no prior surgical history.

On physical examination, the patient had a non-tender, massive right inguinoscrotal hernia that was irreducible. The abdomen was distended and tympanic, with no signs of peritonitis.

**Figure 1:**
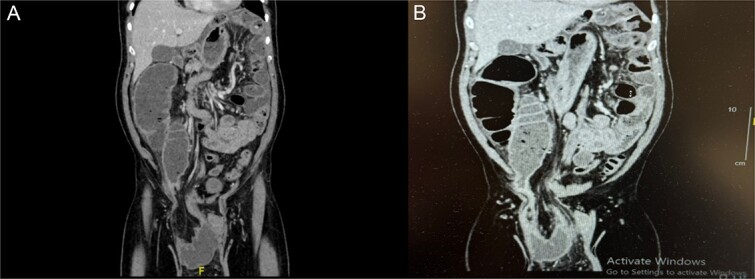
(**A**, **B**) Coronal abdominal CT showing right side inguino-scrotal hernia that contains mesenteric fat and vessels with long segment of transverse colonic loop, there is proximal dilatation of the right side of the colon reaching a competent ileo-cecal valve and not associated with dilatation of the small bowel loops. The left side colon is collapsed.

The patient was admitted for further investigations. Basic routine laboratory tests were unremarkable except for mildly elevated creatinine levels, likely due to dehydration secondary to vomiting and diarrhea. A computed tomography (CT) was ordered due to diagnostic uncertainty given some of the patient’s family members recently suffered from gastroenteritis or if his symptoms were caused by the hernia. The CT scan demonstrated a dilated bowel segment originating from the afferent loop of the TC, which was herniated into the hernia sac and collapsing again at the terminal ileum. The isolated segment showed significant dilation, with a maximum diameter of 6.7 cm. Bowel segments both proximal and distal to this affected segment appeared collapsed, highlighting the transition zone and free fluid was found in the hernia sac and the peri-splenic area (Fig. 1).

**Figure 2:**
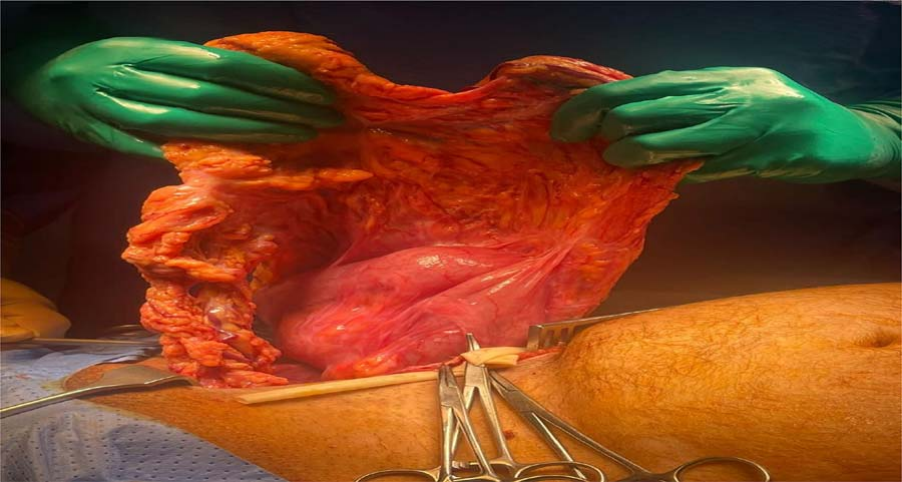
Contents of inguinal hernial sac containing TC and omentum.

Due to the bowel obstruction, the patient was promptly taken to the operating room and booked as an emergent surgery. He underwent an open right inguinal hernia repair under general anesthesia using a modified Shouldice repair technique with mesh. Intraoperatively, the hernia sac was found to contain a viable segment of the TC and omentum which was tethered to apex of the hernia (Fig. 2). After reduction of the contents, the floor was found to have a large weak area, and the floor was reconstructed using a 0 Proline suture in two layers, with the first layer joining the transversalis facia to the posterior rectus sheath and the second joining the shelving edge of the inguinal ligament to the conjoint tendon while reconstructing the internal ring in a modified Shouldice technique. A self-griping polypropylene anatomic mesh was placed and the external oblique, subcutaneous fat, and skin were closed. The patient tolerated the procedure well and was shifted to the post-anesthesia care unit in stable condition.

The patient’s postoperative course was uneventful, his pain was controlled, he was passing bowel movements, and the abdominal distention improved significantly and he was discharged on the fourth postoperative day. At the 1 week and 6 month follow up appointments, the patient reported doing well with no complaints. Physical examination showed a well-healed surgical site with no signs of recurrence or complications.

## Discussion

Herniation of the TC through the inguinal canal, as seen in this case, is extremely rare and noteworthy due to its presentation as a closed-loop bowel obstruction. The two points of obstruction at both the inflow due to a competent ileocecal valve and the outflow at the proximal point of obstruction leads to progressive bowel distension, elevated intraluminal pressure, and a heightened risk of vascular compromise, ischemia, and ultimately perforation if not addressed promptly [1, 4, 8]. Therefore closed loop bowel obstruction is a surgical emergency and needs prompt identification and treatment.

The diagnostic approach to hernias typically begins with physical examination; however, complex hernias, especially those involving bowel obstruction, often require imaging for definitive diagnosis. Ultrasonography, though commonly used, has limitations in sensitivity and specificity for diagnosing complicated hernias. The CT, as employed in this case, offers superior diagnostic utility, providing critical details regarding the hernia’s contents, anatomical extent, and any signs of compromised bowel viability [3]. The use of magnetic resonance imaging, although more sensitive, is often restricted by cost, availability, and the need for sedation in certain patients [10].

The choice of an open approach over laparoscopic was guided by the need for thorough intraoperative evaluation of the herniated bowel segment, given the risk of ischemia inherent in closed-loop obstructions as well as the anticipated adhesions within the hernia sac. While laparoscopic hernia repair is a good surgical option, it may not be suitable for cases requiring reduction of large hernias with expected adhesions and the need for inspection of the hernia contents. The inclusion of mesh in the modified Shouldice repair aimed to enhance the repair’s durability and reduce recurrence risk, aligning with contemporary surgical standards for complex hernia cases [10–12].

The surgical management of this case utilized a modification on the Shouldice repair technique, a well-established surgical approach for inguinal hernia repair that emphasizes the restoration of the inguinal canal’s structural integrity through layered suturing of the posterior wall with superior outcomes [11]. The procedure typically includes excision of the weakened fascia transversalis and reconstruction of the posterior wall of the inguinal canal using a running suture in four layers [11, 12]. The modified Shouldice technique utilizes only the first two layers of the Shouldice repair. This was utilized due to the large weak area in the floor of the inguinal canal which needed reinforcement prior to mesh placement. Mesh repairs, which reduce recurrence rates to ~0.8%, are often preferred for their ability to reinforce the abdominal wall leading to quicker recovery and lower recurrence rates [12].

The outcome of incarcerated hernias with bowel obstruction generally depends on timely intervention and effective management of the hernia and any associated bowel complications. Studies have shown that timely surgical intervention is crucial for preventing severe complications such as bowel necrosis and perforation [10, 12].

Elective hernia repairs generally exhibit a low mortality rate of ~0.1%, whereas emergency procedures have a higher mortality risk of ~3% [13]. Common complications associated with hernia repairs include hematoma formation, wound infection, and injury to surrounding structures, such as blood vessels, bowel, or nerves. These complications are more frequent in older patients and those with multiple comorbidities, though the risk is significantly lower when performed by an experienced surgeon [12–14].

In this case, the timely surgical intervention led to a successful outcome, with no postoperative complications reported. The patient was discharged on the fourth postoperative day and showed well-healed surgical sites at follow-up, underscoring the effectiveness of the selected surgical approach for this complex hernia presentation.

## Conclusion

This report presents a rare case of closed-loop bowel obstruction secondary to TC herniation through the inguinal canal, underscoring the need for heightened clinical vigilance in diagnosing atypical hernia presentations. The use of imaging with diagnostic uncertainty or with complex hernias such as CT scans played a pivotal role in timely diagnosis and facilitating the shift from elective to emergency surgery. Prompt surgical intervention is essential in such cases to prevent severe complications, including bowel ischemia and perforation. This case further supports the need for ongoing vigilance in diagnosing and managing hernias, especially those presenting with atypical symptoms.

## Data Availability

The data used and/or analyzed during the current study are available from the corresponding author on reasonable request.

## References

[1] Aldhafar A, Mohammed A, Alwabari M, et al. A strangulated right inguinal hernia containing the transverse colon: an unusual case report. Asian J Case Rep Surg 2020;3:286–9.

[2] Jensen KK, Henriksen NA, Jorgensen LN. Inguinal hernia epidemiology. In: Hope W, Cobb W, Adrales G (eds). *Textbook of Hernia*. Springer, Cham, 2017, 23–7. 10.1007/978-3-319-43045-4_4.

[3] Ota S, Noguchi T, Takao T, et al. An incarcerated colon inguinal hernia that perforated into the scrotum and exhibited an air-fluid level. Case Rep Med 2015;2015:1–3. 10.1155/2015/105183. PMC4444561 26074967

[4] Lieske B, Meseeha M. Large Bowel Obstruction. [Updated 2024 Nov 9]. In: StatPearls [Internet]. Treasure Island (FL): StatPearls Publishing, 2025 Jan-. Available from: https://www.ncbi.nlm.nih.gov/books/NBK441888/. 28722918

[5] Hassan M, Iqbal U, Fazal MI, et al. Incidence, patterns of presentation and management outcomes of obstructed inguinal hernias presenting to a tertiary care hospital. 2017;11:1235–37, PJMHS.

[6] Smith DA, Kashyap S, Nehring SM. Bowel Obstruction. (Archived). 2023 Jul 31. In: StatPearls [Internet]. Treasure Island (FL): StatPearls Publishing, 2025 Jan-. PMID: 28723004. 28723004

[7] Catena F, De Simone B, Coccolini F, et al. Bowel obstruction: a narrative review for all physicians. World J Emerg Surg 2019;14:20. 10.1186/s13017-019-0240-7. 31168315 PMC6489175

[8] Bali C, Tsironis A, Zikos N, et al. An unusual case of a strangulated right inguinal hernia containing the sigmoid colon. Int J Surg Case Rep 2011;2:53–5. 10.1016/j.ijscr.2011.01.005. 26902552 PMC3284256

[9] Sohrabi C, Mathew G, Maria N, et al. The SCARE 2023 guideline: updating consensus Surgical CAse REport (SCARE) guidelines. Int J Surg Lond Engl 2023;109:1136. 10.1097/JS9.0000000000000373. PMC10389401 37013953

[10] Shakil A, Aparicio K, Barta E, et al. Inguinal hernias: diagnosis and management. Am Fam Physician 2020;102:487–92. 33064426

[11] Chan C, Chan G. The Shouldice technique for the treatment of inguinal hernia. J Minim Access Surg 2006;2:124. 10.4103/0972-9941.27723. 21187981 PMC2999770

[12] Amato B, Moja L, Panico S, et al. Shouldice technique versus other open techniques for inguinal hernia repair. *Cochrane Database of Systematic Reviews* 2012;4:CD001543. 10.1002/14651858.CD001543.pub4. PMC6465190 22513902

[13] Pastorino A, Alshuqayfi A. Strangulated Hernia. [Updated 2022 Dec 19]. In: StatPearls [Internet]. Treasure Island (FL): StatPearls Publishing, 2025 Jan-. Available from: https://www.ncbi.nlm.nih.gov/books/NBK555972/. 32310432

[14] McBee P, Walters R, Fitzgibbons R. Current status of inguinal hernia management: a review. Int J Abdom Wall Hernia Surg 2022;5:159. 10.4103/ijawhs.ijawhs_36_22.

